# Primary Investigation of the Preparation of Nanoparticles by Precipitation

**DOI:** 10.3390/molecules170911067

**Published:** 2012-09-13

**Authors:** Eliska Vaculikova, Veronika Grunwaldova, Vladimir Kral, Jiri Dohnal, Josef Jampilek

**Affiliations:** 1 Faculty of Pharmacy, University of Veterinary and Pharmaceutical Sciences, Palackeho 1/3, 612 42 Brno, Czech Republic; 2 Nanotechnology Centre, VSB—Technical University of Ostrava, 17. listopadu 15/2172, 708 33 Ostrava, Czech Republic; 3 Institute of Chemical Technology, Faculty of Chemical Engineering, Technicka 5, 166 28 Prague 6, Czech Republic; 4 Institute of Inorganic Chemistry, Academy of Science, 25068 Rez, Czech Republic; 5 Research Institute for Pharmacy and Biochemistry, Lidicka 1879/48, 602 00 Brno, Czech Republic

**Keywords:** steroids, nanoparticles, precipitation, excipients, dynamic light scattering

## Abstract

The absorption, distribution, biotransformation and excretion of a drug involve its transport across cell membranes. This process is essential and influenced by the characteristics of the drug, especially its molecular size and shape, solubility at the site of its absorption, relative lipid solubility, *etc*. One of the progressive ways for increasing bioavaibility is a nanoparticle preparation technique. Cholesterol, cholestenolone and pregnenolone acetate as model active pharmaceutical ingredients and some of the commonly used excipients as nanoparticle stabilizers were used in the investigated precipitation method that was modified and simplified and can be used as an effective and an affordable technique for the preparation of nanoparticles. All 120 prepared samples were analyzed by means of dynamic light scattering (Nanophox). The range of the particle size of the determined 100 nanoparticle samples was from 1 nm to 773 nm, whereas 82 samples contained nanoparticles of less than 200 nm. Relationships between solvents and used excipients and their amount are discussed.

## 1. Introduction

For achieve the pharmacological activity of an active pharmaceutical ingredient (API), the solubility of the API in physiological liquids is required, so that the API can be available at the place of absorption. Solubility in various solvents is a characteristic property of a particular compound. The solubility of a compound in water correlates to a great extent with the solubility in physiological liquids and is the first limiting factor for good absorption and biodistribution. From the point of view of pharmaceutical formulations, the solubility of compounds higher than 1% can be considered as satisfactory. When this condition is not met, it is important to improve the solubility. Solubility is not the only important factor; also the solubility rate is essential. This is a physico-chemical property that can be influenced by crystal shape (morphology, polymorphism), particle size, properties of compound surface, *etc*. [1,2,3].

The solubility of an API can be principally influenced in two ways: (i) chemically (salt formation when the molecule is ionizable; other molecule modification to increase hydrophilicity; prodrug preparation); or (ii) by optimization of physico-chemical properties (addition of excipients, particle size reduction or change of polymorphic forms). There are several ways to improve API solubility based on addition of excipients: (i) formation of molecular complexes with solubilizers (e.g., benzoate sodium with caffeine) and/or with soluble salts of polybasic organic acids and hydroxy acids; (ii) generation of inclusion complexes with natural or synthetically modified cyclodextrins or (iii) application of co-solvents (such as ethanol, glycerol, propylene glycol, polyethylene glycol). Solubility can be also increased by addition of surfactants/tensides that create micelles in the aqueous medium. One more frequently used method of solubility increase is complexation of API to native or chemically modified polysaccharide matrixes, for example, of pectins, glucans, chitosans, celluloses, alginates, *etc*. [1,2,3,4,5].

The other possibility how to increase the solubility of an API is preparation of nanoparticles. The advantages of nanotechnology are as follows: (i) increased bioavailability (quick dissolution; improved penetration through membranes); (ii) lower doses; (iii) lower toxicity; (iv) targeted biodistribution; (v) reduction of influence of food on variability; vi) quicker development of formulations [2,6,7,8,9]. Nanoparticles less than 200 nm are of practical importance [10,11,12,13,14,15]. Nevertheless it is necessary to admit some disadvantages of nanoparticles, such as: (i) increased aggregation in biological systems due to high surface energy; (ii) poor solubility and biocompatibility of carbon nanotubes; (iii) short biological half-life due to fast uptake in RES; (iv) high immunogenicity; (v) acute and chronic toxicity, and (vi) irresponsible/unforeseeable safety problems. Especially their possible toxicity comprises a great problem. The toxicity is dependent on the shape and surface properties of nanoparticles, because shape and surface can influence nanoparticle-cell interactions as well as the rate of penetration to cells. From various nanoparticle forms nanotubes were found as one of the most toxic nanoparticle shapes [16,17,18,19].

A wide range of techniques have been developed for the preparation of nanomaterials [8,9,12,13,14,15,20,21,22,23,24,25,26]. Synthetic methods for nanoparticles are typically grouped into two categories: top-down (generally dispergation processes) and bottom-up (generally precipitation processes). The first involves division of a massive solid into smaller portions. This approach may involve milling or attrition, chemical methods and volatilization of a solid followed by condensation of the volatilized components, e.g., high-energy ball milling, high-pressure homogenization, emulsifying technology and microfluidization [13,14,15,20,21,22,23]. The second, bottom-up, method of nanoparticle fabrication involves condensation of atoms or molecular entities in a gas phase or in solution such as sol-gel synthesis [13,14,15,20,24] and precipitation processes, for example, spray freezing into liquid, evaporative precipitation into aqueous solution, precipitation with compressed antisolvent or rapid expansion of supercritical solution) [13,14,15,20,25,26]. The latter approach is by far the most popular in the preparation of nanoparticles.

The aim of this paper is preparation of nanoparticles of cholesterol-like compounds by precipitation. The procedure is in principle similar to the solvent evaporation process, e.g., evaporative precipitation into aqueous solution. Methods based on the similar approach were described recently [27,28,29]. The chosen model APIs represent poorly water soluble compounds. In this pilot screening various types of surface-active agents were investigated. These excipients belong to GRAS substances and were applied in various concentrations. This contribution is the result of our interest in primary screening of nanoparticle preparation. Relationships between a substance, a solvent and a used excipient are discussed.

## 2. Results and Discussion

**Figure 1:**
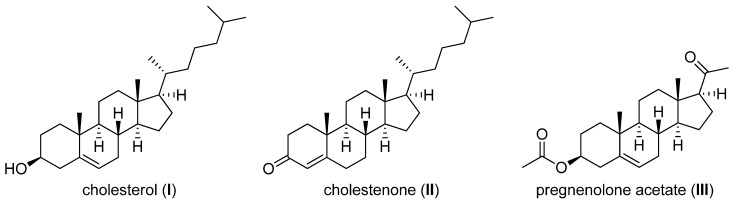
Structures of model APIs.

All three model APIs, cholesterol (5-cholesten-3β-ol, **I**), cholestenolone (4-cholesten-3-one, **II**) and pregnenolone acetate (5-pregnen-3β-ol-20-one acetate, **III**), see Figure 1, were chosen as types of poorly aqueous soluble compounds [4,12].

Used excipients represent various classes of pharmaceutical adjutants that can be utilized as solubility modifying compounds. Tween 80 (polysorbate 80, polyoxyethylene sorbitan monooleate) is a nonionic surfactant and emulsifier. Polyoxyethylene groups are hydrophilic groups, nevertheless C_18_ chain of oleic acid constitutes a lipophilic group. Sodium dodecyl sulfate (SDS, sodium lauryl sulfate) is an anionic surfactant consisting of a C_12_ tail attached to a sulfate group, giving the compound the desired amphiphilic properties. Macrogol 6000 (polyethylene glycol, PEG) is used as an excipient in pharmaceutical formulations. The number represents the average molecular weight of the polyethylene glycol. Carboxymethyl cellulose (CMC, carmellose) is a cellulose derivative with carboxymethyl groups bound to some of the hydroxyl groups of the glucopyranose monomers that make up the cellulose backbone. It is often used as salt-sodium carboxymethyl cellulose (SCMC). It is used as a viscosity modifier or thickener, and to stabilize emulsions in various products. Carboxymethyl dextran (CMD) is a polyanionic derivative of dextran (branched glucan composed of chains of varying lengths). It is supplied as the sodium salt of carboxymethyl dextran (SCMD). It is used as a stabilizer of proteins and other sensitive biopolymers, as a carrier for biosensor surfaces, as a stable non-toxic, non-immunogenic additive and also is used for preparation of low-toxic derivatives with drugs and other pharmacologically active substances [3].

The concentration of excipient was chosen between 1% and 10%. The optimal concentration of surfactant is important for optimal particles wetting. If the concentration is too low, particles float on the surface. If the concentration is too high bubbles appear [30]. The polar acetone (AC) and nonpolar dichloromethane (DCM) were chosen as the most suitable solvents for easy dissolution of the APIs.

**Figure 2:**
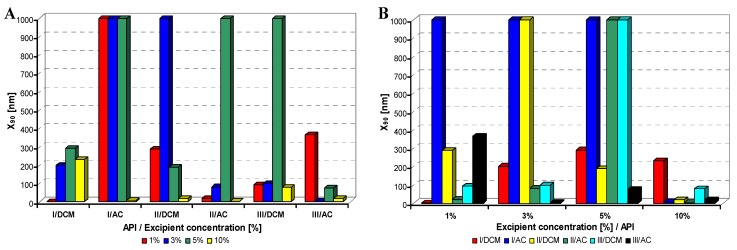
Dependence of particle size (x_90_ [nm]) of model APIs **I**–**III** on concentration [%] of Tween 80 in dichloromethane (DCM) or acetone (AC). (**A**) Samples are grouped according to APIs; (**B**) samples are grouped according to excipient percentage. For clarity sake, the values on y-axis are only to 1,000 nm.

**Figure 3:**
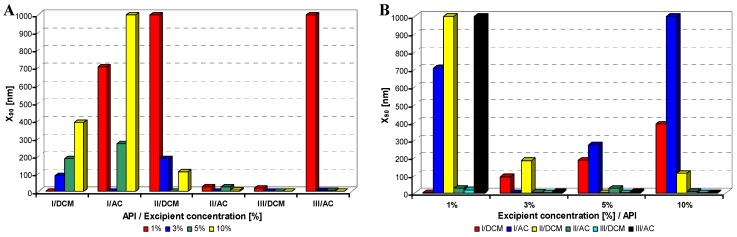
Dependence of particle size (x_90_ [nm]) of model APIs **I**–**III** on concentration [%] of sodium dodecyl sulfate in dichloromethane (DCM) or acetone (AC). (**A**) Samples are grouped according to APIs; (**B**) samples are grouped according to excipient percentage. For clarity sake, the values on y-axis are only to 1,000 nm.

**Figure 4:**
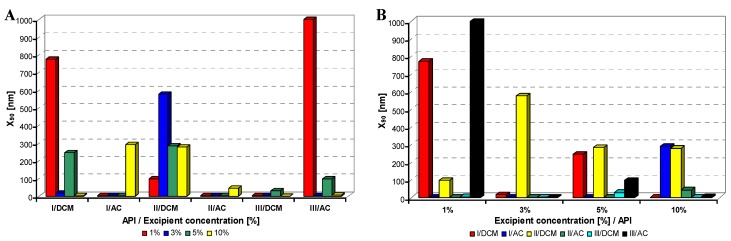
Dependence of particle size (x_90_ [nm]) of model APIs **I**–**III** on concentration [%] of macrogol 6000 in dichloromethane (DCM) or acetone (AC). (**A**) Samples are grouped according to APIs; (**B**) samples are grouped according to excipient percentage. For clarity sake, the values on y-axis are only to 1,000 nm.

**Figure 5:**
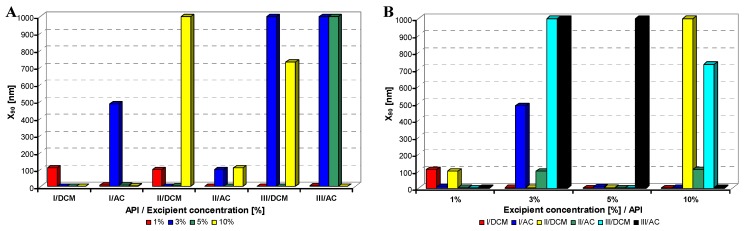
Dependence of particle size (x_90_ [nm]) of model APIs **I**–**III** on concentration [%] of sodium carboxymethyl cellulose in dichloromethane (DCM) or acetone (AC). (**A**) Samples are grouped according to APIs; (**B**) samples are grouped according to excipient percentage. For clarity sake, the values on y-axis are only to 1,000 nm.

**Figure 6:**
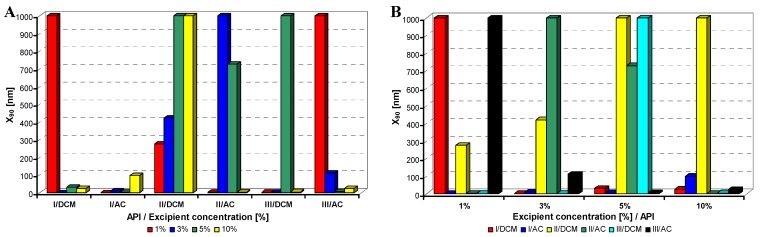
Dependence of particle size (x_90_ [nm]) of model APIs **I**–**III** on concentration [%] of sodium carboxymethyl dextran in dichloromethane (DCM) or acetone (AC). (**A**) Samples are grouped according to APIs; (**B**) samples are grouped according to excipient percentage. For clarity sake, the values on y-axis are only to 1,000 nm.

All model APIs dissolved in dichloromethane and acetone (2% concentration) were added to aqueous solutions (1%, 3%, 5%, 10% concentration) of excipients such as Tween 80 (TW), sodium dodecyl sulfate (SDS), macrogol 6000 (PEG), sodium carboxymethyl cellulose (SCMC) and sodium carboxymethyl dextran (SCMD), *i.e.*, with each excipient 24 samples were prepared. The final relations API: excipient were 1:0.5 (2%:1%), 1:1.5 (2%:3%), 1:2.5 (2%:5%), 1:5 (2%:10%). Samples were obtained by mixing and simultaneous evaporation of organic solvent to final 10 mL sample volume and then characterized by dynamic light scattering [30]. All the results are presented in Table 1, Table 2, Table 3, Table 4 and Table 5 and Figure 2, Figure 3, Figure 4, Figure 5 and Figure 6. Figure 2, Figure 3, Figure 4, Figure 5 and Figure 6 illustrate the dependence of particle size expressed as the cumulative distribution x_90_ [nm] of the compounds **I**–**III** on the concentration [%] of an individual excipient, whereas in Figures A samples are grouped according to individual APIs **I**–**III**, while in Figures B always individual APIs are separated according to the percentage of the excipient. The particle size x_90_ was used for evaluation of the method success, since this value represents 90% of the cumulative particle size distribution in the measured sample.

The dispersity is a measure/degree of the homogeneity/heterogeneity of sizes of particles in a mixture/system. It is possible to see this feature on the width of the particle-size distribution, which is described as differences between cumulative distribution x_10_ and x_90_, see Table 1–Table 5. According to the results, the average relation of the cumulative distribution x_10_/x_90_ ranged from 0.6 to 0.9. It is possible to suppose that nanoparticles are spheres, because the size in dynamic light scattering means the hydrodynamic diameter of the particle. All samples were dispersed by ultrasonics directly before the measurement to avoid possible re-agglomeration. Stabilization of the dispersed samples was achieved by surfactants and by the temperature. The measuring cell was equilibrated at 25 °C, so the Brown motion of nanoparticles is influenced just by their size.

From Figure 2A–Figure 6A it can be stated that particle size is not dependent on model API type but it is strongly influenced by the type and concentration of the utilized excipient. After summarization of all the results it can be concluded that from 120 prepared mixtures 100 samples contained nanoparticles (see Table 1–Table 5), from which 82 samples contained nanoparticles smaller than 200 nm (see Table 1–Table 5, bolded values). Nanoparticles under 10 nm were determined in 51 samples from 82, see Table 1–Table 5 (bolded values with grey background).

**Table 1:** Particle size (x_10_, x_90_ [nm]) of APIs **I**–**III** and concentration [%] of Tween 80 in dichloromethane (DCM) or acetone (AC). All the presented results are reported as medium value of four independent measurements, repeatability was up to 6%. Samples that contained nanoparticles <200 nm are bolded; nanoparticles <10 nm are indicated by grey background. (S.No. = sample number).

**API/Solvent**	**Tween 80**												
	**S.No.**	**1%**		**S.No.**	**3%**		**S.No.**	**5%**		**S.No.**	**10%**		
		x_10_	x_90_		x_10_	x_90_		x_10_	x_90_		x_10_	x_90_	**Particle size [nm]**
**I**/DCM	**1**	1	**1**	**2**	139	**200**	**3**	222	289	**4**	158	231	
**I**/AC	**5**	2038	2242	**6**	9963	10,276	**7**	9344	10,281	**8**	7	**8**	
**II**/DCM	**9**	218	288	**10**	3149	3464	**11**	97	**189**	**12**	18	**19**	
**II**/AC	**13**	14	**21**	**14**	57	**81**	**15**	9345	10,281	**16**	2	**3**	
**III**/DCM	**17**	63	**94**	**18**	91	**99**	**19**	3640	4005	**20**	62	**79**	
**III/**AC	**21**	332	366	**22**	2	**3**	**23**	53	77	**24**	12	**19**	

**Table 2:** Particle size (x_10_, x_90_ [nm]) of APIs **I**–**III** and concentration [%] of sodium dodecyl sulfate in dichloromethane (DCM) or acetone (AC). All the presented results are reported as medium value of four independent measurements, repeatability was up to 6%. Samples that contained nanoparticles <200 nm are bolded; nanoparticles <10 nm are indicated by grey background. (S.No. = sample number).

**API/Solvent**	**Sodium dodecyl sulfate**												
	**S.No.**	**1%**		**S.No.**	**3%**		**S.No.**	**5%**		**S.No.**	**10%**		
		x_10_	x_90_		x_10_	x_90_		x_10_	x_90_		x_10_	x_90_	**Particle size [nm]**
**I**/DCM	**25**	1	**2**	**26**	61	**91**	**27**	136	**186**	**28**	286	391	
**I**/AC	**29**	503	706	**30**	1	**1**	**31**	195	272	**32**	9344	10,280	
**II**/DCM	**33**	9345	10,281	**34**	134	**184**	**35**	1	**2**	**36**	1001	**111**	
**II**/AC	**37**	24	**26**	**38**	2	**3**	**39**	18	**27**	**40**	6	**9**	
**III**/DCM	**41**	12	**18**	**42**	1	**2**	**43**	1	**2**	**44**	1	**2**	
**III**/AC	**45**	771	1083	**46**	4	**7**	**47**	4	**7**	**48**	1	**2**	

**Table 3:** Particle size (x_10_, x_90_ [nm]) of APIs **I**–**III** and concentration [%] of macrogol 6000 in dichloromethane (DCM) or acetone (AC). All the presented results are reported as medium value of four independent measurements, repeatability was up to 6%. Samples that contained nanoparticles <200 nm are bolded; nanoparticles <10 nm are indicated by grey background. (S.No. = sample number).

**API/Solvent**	**Macrogol 6000**												
	**S.No.**	**1%**		**S.No.**	**3%**		**S.No.**	**5%**		**S.No.**	**10%**		
		x_10_	x_90_		x_10_	x_90_		x_10_	x_90_		x_10_	x_90_	**Particle size [nm]**
***I***/DCM	**49**	534	773	**50**	15	**16.86**	**51**	179	247	**52**	1	**2**	
***I***/AC	**53**	2	**2**	**54**	3	**4**	**55**	2	**3**	**56**	193	292	
***II***/DCM	**57**	90	**99**	**58**	408	576.85	**59**	197	285	**60**	211	279	
***II***/AC	**61**	1	**2**	**62**	1	**2.27**	**63**	1	**2**	**64**	29	**44**	
***III***/DCM	**65**	3	**4**	**66**	2	**3**	**67**	27	**30**	**68**	1	**1**	
***III***/AC	**69**	2929	3222	**70**	3	**3**	**71**	90	**99**	**72**	6	**6**	

**Table 4:** Particle size (x_10_, x_90_ [nm]) of APIs **I**–**III** and concentration [%] of sodium carboxymethyl cellulose in dichloromethane (DCM) or acetone (AC). All the presented results are reported as medium value of four independent measurements, repeatability was up to 6%. Samples that contained nanoparticles <200 nm are bolded; nanoparticles <10 nm are indicated by grey background. (S.No. = sample number).

**API/Solvent**	**Sodium carboxymethyl cellulose**												
	**S.No.**	**1%**		**S.No.**	**3%**		**S.No.**	**5%**		**S.No.**	**10%**		
		x_10_	x_90_		x_10_	x_90_		x_10_	x_90_		x_10_	x_90_	**Particle size [nm]**
**I**/DCM	**73**	101	**111**	**74**	2	**3**	**75**	1	**1**	**76**	1	**2**	
**I**/AC	**77**	6	**9**	**78**	357	486	**79**	7	**9**	**80**	2	**3**	
**II**/DCM	**81**	90	**99**	**82**	1	**2**	**83**	3	**3**	**84**	879	1249	
**II**/AC	**85**	2	**3**	**86**	90	**99**	**87**	1	**2**	**88**	101	**111**	
**III**/DCM	**89**	1	**2**	**90**	8799	9987	**91**	1	**2**	**92**	534	731	
**III**/AC	**93**	4	**6**	**94**	9345	10,281	**95**	9345	10,281	**96**	2	2	

**Table 5:** Particle size (x_10_, x_90_ [nm]) of APIs **I**–**III** and concentration [%] of sodium carboxymethyl dextran in dichloromethane (DCM) or acetone (AC). All the presented results are reported as medium value of four independent measurements, repeatability was up to 6%. Samples Samples that contained nanoparticles <200 nm are bolded; nanoparticles <10 nm are indicated by grey background. (S.No. = sample number).

**API/Solvent**	**Sodium carboxymethyl dextran**												
	**S.No.**	**1%**		**S.No.**	**3%**		**S.No.**	**5%**		**S.No.**	**10%**		
		x_10_	x_90_		x_10_	x_90_		x_10_	x_90_		x_10_	x_90_	**Particle size [nm]**
**I**/DCM	**97**	5862	8981	**98**	1	**1**	**99**	20	**31**	**100**	17	**26**	
**I**/AC	**101**	1	**1**	**102**	8	**9**	**103**	3	**4**	**104**	90	**99**	
**II**/DCM	**105**	209	275	**106**	354	422	**107**	9345	10,281	**108**	2022	3205	
**II**/AC	**109**	3	**3**	**110**	9345	10,281	**111**	535	729	**112**	1	**2**	
**III**/DCM	**113**	1	**1**	**114**	1	**2**	**115**	1418	1560	**116**	3	**5**	
**III**/AC	**117**	9344	10,281	**118**	101	**111**	**119**	5	**8**	**120**	22	**24**	

Generally it can be stated that macrogol provided mostly nanoparticles, and sodium dodecyl sulfate as well as sodium carboxymethyl cellulose afforded most of nanoparticles under 200 nm; the latter yielded most of nanoparticles less than 10 nm.

Table 6 summarizes results of all the samples of nanoparticles under 900 nm size depending on solvents and the type and amount of excipients. As the aim of this contribution is specification of suitable conditions for nanoparticles preparation, in Table 6 generated nanoparticles are not divided according to used APIs.

**Table 6:** View of formed samples of nanoparticles (≤900 nm) depending on solvents and type and amount of excipients. (conc. = concentration, excp. = excipient, dichloromethane = DCM, acetone = AC, Tween 80 = TW, sodium dodecyl sulfate = SDS, macrogol 6000 = PEG, sodium carboxymethyl cellulose = SCMC, sodium carboxymethyl dextran = SCMD).

**Excp. conc./type**	**DCM**				**Sum total**	**Overall average** x_90_**[nm]**	**AC**				**Sum total**	**Overall average** x_90_**[nm]**
	**1%**	**3%**	**5%**	**10%**			**1%**	**3%**	**5%**	**10%**		
	number of nanop. samples						number of nanop. samples					
TW	3	2	2	3	10	149	2	2	1	3	8	72
SDS	2	3	3	3	11	90	2	3	3	2	10	106
PEG	3	3	3	3	12	193	2	3	3	3	11	42
SCMC	3	2	3	2	10	96	3	2	2	3	10	73
SCMD	2	3	1	2	8	95	2	2	3	3	10	99
**Sum total**	13	13	12	13	51	623	11	12	12	14	49	392
**Overall average**	136	123	106	145	510	125	104	68	103	45	320	78
						127						80

In Table 6 results of all the determined nanoparticles are listed. Based on these results it can be stated that acetone as polar solvent is more advantageous for nanoparticle generation in case of Tween, macrogol and sodium carboxymethyl cellulose (significantly less nanoparticle size average (calculated using x_90_): 72, 42, 73 nm compared with DCM and approximately the same number of nanoparticle samples). In case of sodium carboxymethyl dextran the number of nanoparticle samples and their nanoparticle size were approximately the same as when using acetone and dichloromethane (10-99/8-95). Dichloromethane seems to be more advantageous only in combination with sodium dodecyl sulphate, where smaller nanoparticles were determined (10-106/11-90).

If the influence of excipient concentration in acetone and dichloromethane is considered, it can be stated that generally 10% and 3% concentrations of excipient in acetone, *i.e.*, API:excipient ratio 1:5 and 1:1.5, or 5% and 3% concentrations of excipient in dichloromethane, *i.e.*, API:excipient ratio 1:2.5 and 1:1.5, seem to be the most advantageous for maximum number of nanoparticle samples with the smallest nanoparticle size, see Table 6 and Figure 2B–Figure 6B. If amounts of excipients regardless organic solvents are considered, the most favourable concentrations are the following: sodium dodecyl sulphate 3% (API:excipient ratio 1:1.5) and 5% (API:excipient ratio 1:2.5), sodium carboxymethyl cellulose 5% (API:excipient ratio 1:2.5), Tween and sodium carboxymethyl dextran 10% (API:excipient ratio 1: 5) and macrogol 3–10% (API:excipient ratios 1:1.5–5).

Based on the above discussed facts it can be concluded that macrogol 6000, sodium dodecyl sulphate or sodium carboxymethyl cellulose can be used as effective nanoparticle-stabilizing agents in API:excipient ratios 1:1.5, 1:2.5, 1:5. The polar solvent acetone is preferable to nonpolar dichloromethane, probably due to the fact that acetone has higher boiling point (b.p. 56 °C) than dichloromethane (b.p. 39 °C) and it evaporates more slowly in comparison with dichloromethane (dynamically and/or kinetically controlled precipitation). During slow evaporation of organic solvent formed API particles could be more effectively stabilized by excipients in nanoparticle size (dynamic process of nanoparticles generation). Results with APIs dissolved in acetone provided the number of nanoparticle samples comparable with dichloromethane (49/51), but the particle size of APIs dissolved in acetone was smaller than that of APIs dissolved in dichloromethane by third (392/623).

## 3. Experimental

### 3.1. General

All substances as well as excipients were purchased from Sigma-Aldrich (Prague, Czech Republic). Dichloromethane was purchased from Merck (Darmstadt, Germany). Acetone was purchased from LachNer (Neratovice, Czech Republic). All compounds as well as solvents were of analytical grade. H_2_O-HPLC—Mili-Q Grade was used as a solvent of excipients. Particle sizes of all the final samples were determined using dynamic light scattering in a Sympatec Photon Cross-correlation Sensor Nanophox (Sympatec GmbH, System-Partikel-Technik, Clausthal-Zellerfeld, Germany), He-Ne laser 632.8 μm, intensity max. 10 mW. The measuring cell was equilibrated at 25 °C.

### 3.2. Synthesis

#### 3.2.1. Standardized General Procedure for Preparation of Nanoparticles

Tween 80, sodium dodecyl sulfate (SDS), macrogol 6000 (PEG), sodium carboxymethyl cellulose (SCMC) and sodium carboxymethyl dextran (SCMD) were used as excipients. Each excipient (0.1 g, 0.3 g, 0.5 g or 1.0 g) was dissolved in water (10 mL), and four solutions with concentrations 1%, 3%, 5% and 10% were prepared. Cholesterol, cholestenolone and pregnenolone acetate (0.2 g) were dissolved in dichloromethane or acetone (10 mL), *i.e.*, 2% solutions were prepared. The solutions of the substances in dichloromethane (DCM) or acetone (AC) were slowly dropped (2 mL/min) to the aqueous solutions of excipients that were stirred (600 rpm). Then the system was stirred (600 rpm) for 10 min at 35 °C, after which the mixtures were transferred to an ultrasonic bath in the fume chamber, where they were mixed again for 40 min, and simultaneously organic solvent was evaporated. The final volume of the aqueous sample was 10 mL. The particle size of nanonized substances in samples was evaluated by means of Nanophox. All samples were dispersed by ultrasonics directly before the measurement. Measurements were repeated four times. All presented results are reported as medium value of these independent measurements. Repeatability was up to 6%. The results are summarized in Table 1–Table 5 and illustrated in Figure 2–Figure 6.

## 4. Conclusions

One hundred and twenty samples of cholesterol (**I**), cholestenolone (**II**) and pregnenolone acetate (**III**) were prepared by precipitation in media Tween 80, sodium dodecyl sulfate, macrogol 6000, sodium carboxymethyl cellulose and sodium carboxymethyl dextran. All the samples were analyzed by a Nanophox spectrometer. According to the cumulative distribution x_90_, 100 samples contained nanoparticles; 82 samples contained nanoparticles <200 nm; and 51 samples contained nanoparticles <10 nm. The smallest nanoparticle was 1 nm, the largest size was 773 nm. The polar solvent acetone was more preferable than nonpolar dichloromethane. Sodium dodecyl sufate, sodium carboxymethyl cellulose and macrogol 6000 in concentrations 10% and 3%, *i.e.*, API:excipient ratios 1:5, 1:1.5, possessed the most advantageous nanoparticle-stabilizing properties. It can be concluded that the investigated precipitation method can be used as an effective and an affordable technique for the preparation of nanoparticles. The selected conditions are convenient for formation of nanoparticles, and the used excipients are principally applicable as nanoparticle stabilizers.
